# Functional modules of sigma factor regulons guarantee adaptability and evolvability

**DOI:** 10.1038/srep22212

**Published:** 2016-02-26

**Authors:** Sebastian C. Binder, Denitsa Eckweiler, Sebastian Schulz, Agata Bielecka, Tanja Nicolai, Raimo Franke, Susanne Häussler, Michael Meyer-Hermann

**Affiliations:** 1 Department of Systems Immunology and Braunschweig Integrated Centre of Systems Biology, Helmholtz Centre for Infection Research, 38124 Braunschweig, Germany; 2 Institute for Molecular Bacteriology, TWINCORE GmbH, Center for Clinical and Experimental Infection Research, a joint venture of the Hannover Medical School and the Helmholtz Center for Infection Research, 30265 Hannover, Germany; 3 Department of Molecular Bacteriology, Helmholtz Centre for Infection Research, 38124 Braunschweig, Germany; 4 Department of Chemical Biology, Helmholtz Centre for Infection Research, 38124 Braunschweig, Germany; 5 Institute for Biochemistry, Biotechnology and Bioinformatics, Technische Universität Braunschweig, 38124 Braunschweig, Germany

## Abstract

The focus of modern molecular biology turns from assigning functions to individual genes towards understanding the expression and regulation of complex sets of molecules. Here, we provide evidence that alternative sigma factor regulons in the pathogen *Pseudomonas aeruginosa* largely represent insulated functional modules which provide a critical level of biological organization involved in general adaptation and survival processes. Analysis of the operational state of the sigma factor network revealed that transcription factors functionally couple the sigma factor regulons and significantly modulate the transcription levels in the face of challenging environments. The threshold quality of newly evolved transcription factors was reached faster and more robustly in *in silico* testing when the structural organization of sigma factor networks was taken into account. These results indicate that the modular structures of alternative sigma factor regulons provide *P. aeruginosa* with a robust framework to function adequately in its environment and at the same time facilitate evolutionary change. Our data support the view that widespread modularity guarantees robustness of biological networks and is a key driver of evolvability.

Controlling the rate of gene transcription is a fundamental biological process, ultimately dictating the cellular phenotype and bacterial adaptive processes to diverse environments^1^. *Pseudomonas aeruginosa* is a Gram-negative bacterium that can be found in various and challenging habitats^2^. It is not only an adaptive environmental bacterium but also an important opportunistic pathogen which causes devastating acute and chronic persistent infections^3^,^4^,^5^ and exhibits an extremely broad host range^6^,^7^. The main reason for the ecological success of *P. aeruginosa* can be attributed to its large metabolic versatility and environment-driven flexible changes in the transcriptional profile. Sequencing of the *P. aeruginosa* reference strains revealed a large genome with highly abundant global regulators and signaling systems that form a complex and dynamic regulatory network^8^. Among transcriptional regulators, sigma factors (more than 25 of which have been described in *P. aeruginosa*) are of exceptional importance as they provide promoter recognition specificity to the RNA polymerase core enzyme and mediate cellular responses to environmental cues through redirection of transcription initiation^9^,^10^,^11^,^12^.

A long-standing question in biology is how populations are capable of adapting to novel and challenging environments and thus conquer new niches. In this context, it has been repeatedly argued^13^ that the modularity of the underlying developmental systems is key to the ability to evolve. By shifting from molecular to modular cell biology general principles are expected to be uncovered of how cells robustly detect and amplify signals in a noisy environment and at the same time evolve by genetic changes to adapt to new challenges^13^. We have recently demonstrated that in *P. aeruginosa* alternative sigma factor regulons are discrete functional modules that exhibit only limited overlap in their transcriptional response to external stimuli^14^. Whereas a self-contained activity of the various alternative sigma factor-dependent functional modules would guarantee robustness and maintenance in a noisy environment, bacterial adaptation to new and challenging habitats might be reflected by connectivity among the regulons. Here, to follow on this hypothesis, we analyzed the functional and operational status of the sigma factor networks in the opportunistic pathogen *P. aeruginosa*. By linking experimental data with *in silico* testing we uncovered a general principle of how one of the largest bacterial genomes is structured. We argue that connectivity among the subunits of functional sparsely connected alternative sigma factor governed modules via global transcription factors enable *P. aeruginosa* to reconcile robustness and flexibility to a large variety of resources and habitats.

## Results

### Functional status of alternative sigma factor regulons

**Figure 1:**
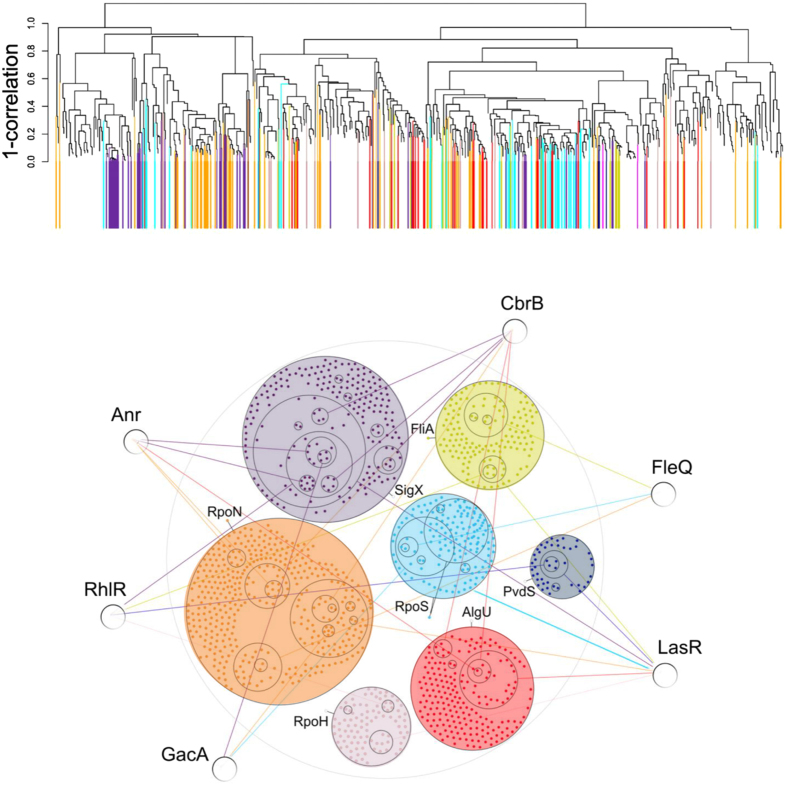
Activation of transcription factors provides connectivity among the functional modules of the alternative sigma factors. (**a**) Hierarchical clustering tree summarizing the co-expression patterns of genes previously identified as differentially regulated under changing environmental conditions^15^. Genes were clustered applying the average linkage rule on the pair-wise Pearson correlation between their normalized expression values (for further details please see Materials and Methods). Genes that have been assigned to a single alternative sigma factor primary regulon (305 genes)^14^ are depicted in color. Genes that were ambiguously assigned are depicted in white (491 genes), (**b**) Power graph presentation of the connectivity of alternative sigma factor regulons via global transcription factors. Genes are shown as colored dots within the colored circles defining the RpoH, FliA, SigX, PvdS, AlgU, RpoS and RpoN alternative sigma factor regulons. The six global transcription factors (CbrB, GacA, Anr, FleQ, RhlR, and LasR) regulate subsets of genes within the sigma factor regulons as shown with colored connectors to likewise encircled genes. The radius of the circles reflects the number of genes within the respective sigma factor regulons.

By defining the genomic suite of alternative sigma factor binding sites throughout the *P. aeruginosa* genome it became apparent that alternative sigma factor regulons are discrete functional modules that exhibit only limited overlap^14^. While this may provide stable responses to external stimuli that are sensed by the various alternative sigma factors, we became interested in the functional states of sigma factors and how their regulons are expressed under flexible experimental conditions. The transcriptional profiles of the *P. aeruginosa* type strain PA14 grown under a plethora of different environmental conditions have been analyzed previously^15^. These included growth within biofilms, at various temperatures, osmolarities and phosphate concentrations, under anaerobic conditions, attached to a surface and conditions encountered within the eukaryotic host. Among the 796 genes that were found to be differentially regulated between at least two of the 14 tested environmental conditions (adaptive transcriptome^15^), 305 have been assigned unambiguously to a specific alternative sigma factor regulon^14^, colored in Fig. 1a. If the alternative sigma factor regulons would be insulated functional modules, one would expect that genes of the various regulons would be largely co-regulated even under flexible environmental conditions as long as those conditions activate the respective alternative sigma factor. Previously, transcriptional profiling of *Bacillus subtilis* under various environmental conditions indeed uncovered a modular expression structure that was reflecting sigma factor regulons^16^. In this study, clearly, co-expression patterns of genes affected by the seven major *P. aeruginosa* alternative sigma factors (RpoN, RpoS, RpoH, AlgU, SigX, FliA, PvdS) were observed within the adaptive transcriptome (Fig. 1). However, clustering of genes of the various alternative sigma factor regulons was not prominent, indicating that under complex environmental conditions, sub-sets of genes of different alternative sigma factor regulons were co-regulated and became activated simultaneously.

### Transcription factor regulated genes show an enrichment of genes belonging to distinct alternative sigma factor regulons

The activation of a discrete sub-set of genes is a well-known adaptation response of *P. aeruginosa* to complex and changing habitats and is commonly affected by the activity of not only alternative sigma factors but also transcription factors. The genome of *P. aeruginosa* strain PA14 contains more than 6000 genes, 521 of which are annotated as transcriptional regulators. We found altogether 43 transcription factors to be differentially expressed within the adaptive transcriptome^15^. For the majority, 33, of those we could not identify an unambiguous assignment to one of the alternative sigma factor regulons^14^. These are most likely regulated by the housekeeping sigma factor RpoD. This is interesting since it supports the finding that alternative sigma factor regulons exhibit only limited direct cross-talk. RpoD governed transcription factors do not contribute to direct cross-talk since they do not link an inducing alternative sigma factor to the expression of genes belonging to a second alternative sigma factor regulon.

To analyze which genes are affected by global *P. aeruginosa* transcription factors and to uncover whether they contribute to connectivity of the alternative sigma factor regulons, we selected six transcription factors (CbrB, GacA, Anr, FleQ, RhlR, and LasR), all of which are known to regulate large numbers of genes. We recorded the transcriptional profiles of the respective mutants of the Harvard Medical School PA14 transposon mutant library^17^. Remarkably, we found in each experimentally determined transcriptional profile a preference of genes belonging to a specific subset of alternative sigma factor regulons (Fig. 1b). We compared the enrichment of genes belonging to specific alternative sigma factor regulons in the transcriptional profile of the transcription factor relative to their overall abundance in the *P. aeruginosa* genome by calculating an enrichment factor (EF) (details in Materials and Methods). Interestingly, we found an enrichment of RpoS-controlled genes in the regulon of the transcription factor LasR (EF = 1.63) consistent with previous studies which uncovered the contribution of RpoS, LasR-LasI and RhlR-RhlI to the complex architecture of the quorum sensing regulon in *P. aeruginosa*^18^,^19^,^20^,^21^. Likewise, the FleQ regulon shows preference for FliA (EF = 3.44) and RpoN (EF = 1.22) regulated genes which is in line with their known function: a comprehensive analysis of the flagellar biosynthesis in *P. aeruginosa* revealed FleQ and RpoN on top of the regulatory cascade, while FliA is required for the expression of effector genes such as *fliC-fleL, cheAB-motAB-cheW, cheVR, flgMN* and *cheYZ*^22^. The global response regulator GacA has been found to preferentially modulate the expression of genes which are under the guidance of RpoS (EF = 1.67). Previously GacA was found to control hydrogen cyanide biosynthesis via the transcriptional control of the quorum-sensing gene *rhlI*^23^. Moreover, GacA and RpoS have been shown to be involved in biofilm formation, indicating a functional link of these global regulators^24^,^25^. The composition analysis of the CbrB regulon revealed an over-representation of genes which were under control of RpoN (EF = 1.27). This finding is in accordance with previous results that link the CbrA/CbrB two-component regulatory system to the regulation of the utilization of multiple carbon and nitrogen sources in *P. aeruginosa*^26^. Further studies could show that the regulation of carbon and nitrogen utilization is coordinated by a network of the two-component systems CbrAB and NtrBC^27^. Additionally, the CbrA/CbrB system is involved in metabolism, virulence and antibiotic resistance in *P. aeruginosa*^28^ which is consistent with the numerous functions and the mode of action of RpoN^29^,^30^,^31^,^32^,^33^,^34^,^35^,^36^. Interestingly, SigX target genes were identified to be over-represented in the regulons of the transcription factors Anr (EF = 1.68), CbrB (EF = 1.30) and RhlR (EF = 2.12). These results underline the significance of this under-estimated and recently in more detail characterized sigma factor^37^,^38^,^39^,^40^.

In conclusion, the composition of global transcription factor profiles in respect to the affiliation of their genes to specific alternative sigma factor regulons is i) transcription factor specific and ii) does not reflect the overall composition of the alternative sigma factor dependent genes throughout the genome. These results imply that by creating connectivity among alternative sigma factor regulons the transcription factors govern a distinct composition of genes, which has been selected from only a sub-fraction of well approved composite genes of the genome and rely on the well proven, combat-ready alternative sigma factor functional modules.

### Evolution of new transcription factors is facilitated by the modular structure of alternative sigma factor regulons

Incremental changes in coding and non-coding sequences are the key drivers of genome evolution and allow for adaptations to new and challenging environmental conditions. However, it has been assumed that especially those genes which exhibit central and pleiotropic functions experience a great deal of evolutionary limits and constraints. It thus might be expected that alternative sigma factors have undergone stabilizing selection, and are therefore conserved and limited in their evolutionary response to future environmental changes.

Genome evolution can also be driven by the emergence of new genes. There is a growing interest in novel taxonomically restricted genes that are free to evolve new functions without suffering from the constraining effect of pleiotropy. New genes commonly arise through the duplication of existing genes and may maintain similar functions to the parental gene over a long evolutionary period or may undergo a process of diversification until a completely new function is evolved.

We hypothesized that the organization of the genome in distinct alternative sigma factor governed structural modules which govern pleiotropic phenotypes limits the space for the evolution of alternative sigma factors but facilitates the evolution of novel transcription factor regulons that create connectivity between the alternative sigma factor regulons in a way that allows organisms to adapt to new challenges.

We therefore determined the sequence variation within the coding sequence of the ten major alternative sigma factors (RpoN, RpoS, RpoH, AlgU, SigX, FliA, PvdS, FecI, FecI2, and FpvI) and the housekeeping sigma factor RpoD as well as of the six global transcription factors (CbrB, GacA, Anr, FleQ, RhlR, and LasR) across the previously profiled 151 clinical *P. aeruginosa* strains^15^. Indeed, the overall sequence variation was lower for those genes encoding sigma factors as compared to those encoding transcription factors. The median of the sums of nucleotide positions exhibiting nonsynonymous single nucleotide polymorphisms (SNPs) in at least one of the 151 clinical isolates, normalized to the gene length, was 3.78% versus 5.84%, and thus was significantly lower in the genes encoding sigma factors (Wilcoxon rank sum test, p = 0.01). Sequence variation was even lower in sub-regions which correspond to DNA-binding domains. This has been observed before^41^,^42^ and indicates that there is limiting space for the evolution of sigma factors. In contrast, coding sequence variations within the six global transcriptional regulators (CbrB, GacA, Anr, FleQ, RhlR, and LasR) were more frequent implicating that the transcriptional regulators can evolve new functions and may drive the evolution of connectivity among sigma factor regulons.

**Figure 2:**
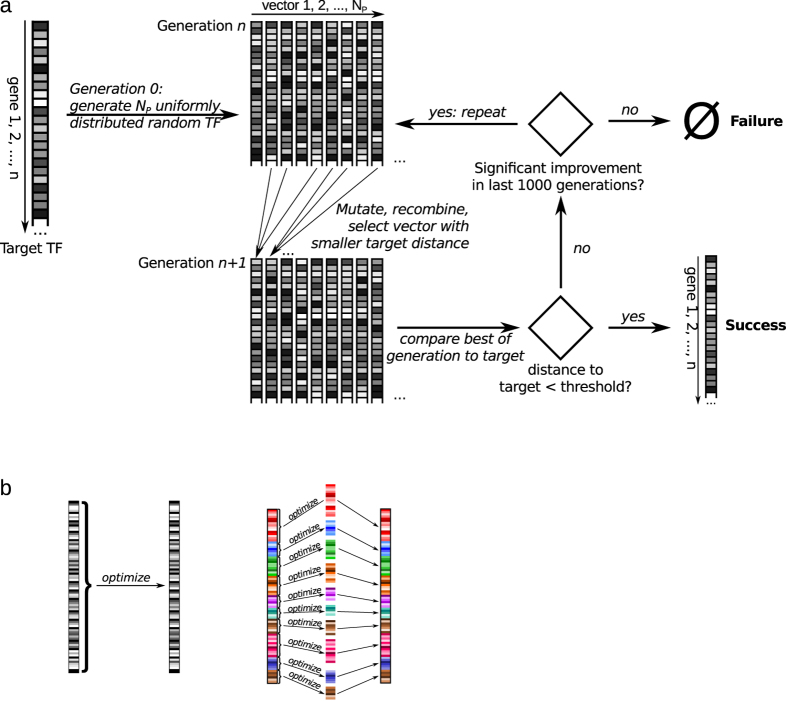
Evolution of a new transcription factor *in silico*. (**a**) Overview of the simulated evolution of a hypothetical target transcription factor. A population of numerical vectors with values between 0 and 1 (indicated by the grey shade) representing gene expression values is randomly generated and evolved over subsequent generations until termination criteria indicated in the flowchart are met, (**b**) Optimization takes place either once in the whole genome (left) or repeatedly within individual sigma factors (right). Colors indicate modular units (sigma factors) for the optimization process and different shades of the same color the expression level.

**Figure 3:**
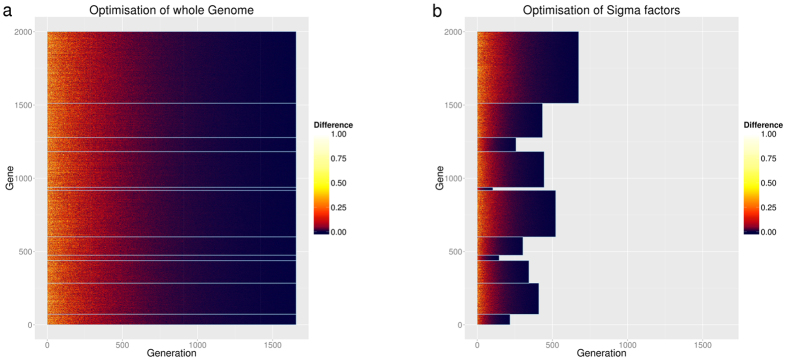
Heat maps of the evolution towards a randomly chosen target transcription factor on a genome of 2000 genes structured by 11 sigma factors of random size. (**a**) simultaneous evolution of the whole genome, (**b**) parallel evolution within sigma factors. Colors from yellow to blue encode the quality of the best evolved transcription factor in each generation. Evolution is stopped when the target transcription factor is found.

**Figure 4:**
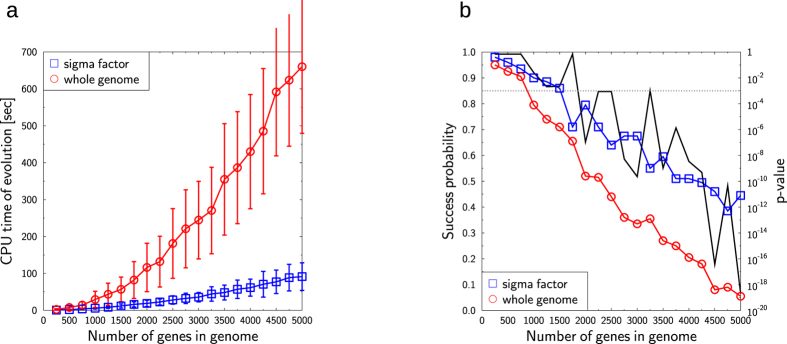
Evolution of a new transcription factor *in silico*. (**a**) CPU time required to evolve a new transcription factor *in silico* for different genome sizes when evolution is based on the whole genome (red) or on sigma factors (blue). Error bars indicate standard deviation in 200 simulations, (**b**) probability of success to find the target transcription factor *in silico* for different genome sizes when evolution is based on whole genome (red) or sigma factors (blue). P values are provided (black line, right axis) for the difference between both strategies.

To test whether the organization of the genome provides a level of biological organization that is critical for the evolution of new transcription factors, we simulated the generation of an optimized transcription factor (represented as a set of gene expression levels between 0 and 1) by using an evolutionary algorithm^43^ that attempts to approach the target transcription factor by evolving a population of NP randomly chosen transcription factors over multiple offspring generations. New generations are created by mutating and recombining transcription factors from the parent generation and selecting those individuals for the filial generation that are closer to the optimal transcription factor (Fig. 2a). To analyze the influence of sigma factors on the speed of finding the target transcription factor, optimization was performed either once across the whole genome or by repeatedly optimizing across multiple subsets that cover the whole genome and correspond to the sigma factors (Fig. 2b). We found that the target transcription factor was evolved in substantially fewer generations when the structural organization of sigma factor networks was taken into account (Fig. 3). Accordingly, the CPU time required to find the optimal transcription factor was significantly reduced if the search was based on organization of genes within sigma factor regulons (Fig. 4a). With increasing size of the genome, the CPU time required for the evolution of new transcription factors dramatically increases (faster than polynomial) when based on whole genome optimization, while it increases only moderately if based on sigma factor regulons (Fig. 4a). The probability of actually finding a new transcription factor by evolution is significantly higher when based on sigma factor regulons (Fig. 4b), suggesting that organization of the genome in sigma factors makes the evolution of new transcription factors not only faster but also more stable. The speed of finding a newly evolved transcription factor could be increased further if their respective regulons were restricted to genes belonging to only a subset of the sigma factor regulons (Fig. S1). Thus, our finding of a relative enrichment of genes belonging to a specific subset of sigma factor regulons in each of the experimentally determined transcription factor regulons is in accordance with the *in silico* predictions. These results imply that evolution of transcription factors is accelerated and facilitated by the organization of the genome in functional modules and is further reinforced if the newly evolved transcription factor regulon resorts to a selected choice of genes within distinct alternative sigma factor regulons. This result is remarkable as in most systems speed and stability of evolutionary processes are mutually exclusive^44^.

### Evolutionary advantages of the modular organization are largely independent of the regulon

The *in silico* simulations predicting increased robustness and speed of the evolution of new alternative sigma factors have been derived from simulations with purely random transcription factors. They could show a structural advantage of the modular organization in alternative sigma factors without assuming a particular structure of the regulons. To test whether or not this advantage is also observable in the six transcription factors whose transcriptional profiles and sigma factor association were studied here, the same simulations were repeated with the experimentally determined gene expression values, regulons, and sigma factor usage for all six transcription factors. Despite the varying number of genes in the different regulons, the simulation results were comparable in all cases and clearly showed an evolutionary advantage in all cases (Fig. S2).

To further test the influence of the regulon size and of a bias in selection of genes from particular sigma factors systematically, hypothetical transcription factors with regulon sizes of 30, 200, 400, 800 and 1200 genes were generated. Genes were attributed to these regulons either by randomly choosing from all genes with equal probabilities (Fig. S3, green curve) or by imposing a preference for small (Fig. S3, blue curve) or large (Fig. S3, red curve) sigma factors on the selection process. The efficiency of the evolutionary optimization process was evaluated by comparing the number of generations, Δ*n*, to reach a threshold in the mean distance between the hypothetical transcription factor target and the best candidate in each generation.

**Figure 5:**
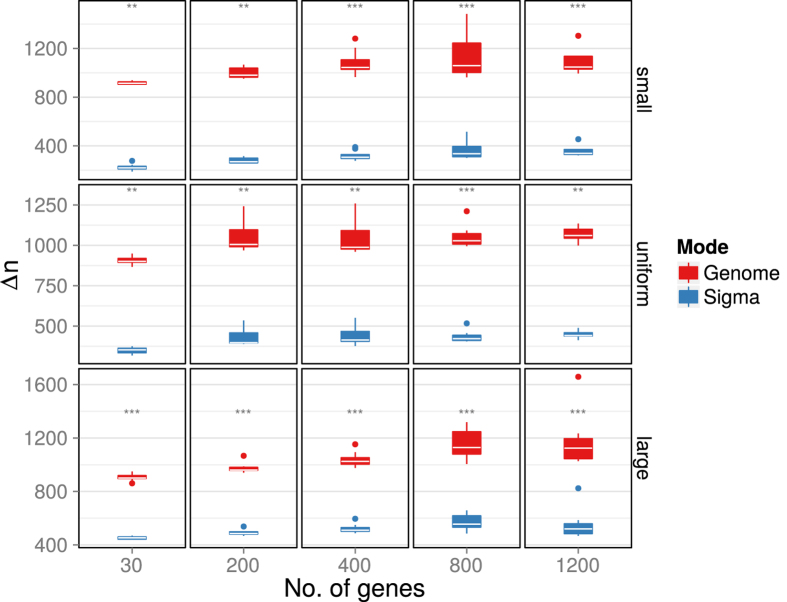
Generation number Δ*n* required to reach a threshold quality index of 0.01 in randomly generated transcription factors. Each boxplot represents results from simulations with ten different randomly generated transcription factors. Different regulon sizes were used as indicated on the horizontal axis. Upper row: genes from small sigma factors were preferred in the generation of transcription factors, middle row: genes were randomly chosen without bias, bottom row: genes from large sigma factors were preferred. Asterisks indicate significance levels as determined by the Mann-Whitney test and corrected for multiple comparisons; ***p < 0.001, **p < 0.005, *p < 0.05.

The advantage of the evolution in sigma factors in terms of evolutionary optimization efficiency is robustly visible in all cases (Fig. 5). The time to reach the threshold quality is significantly shorter when optimizing in individual sigma factors instead of the whole genome at once. This finding is independent of the regulon size (columns in Fig. 5) and of a preference for small or large sigma factors (Fig. 5, rows). Interestingly, although selection and algorithm termination in the simulations were based on the whole transcriptome and the dimensionality of the optimization problem was hence constant in all simulations, very small transcription factor regulons showed a trend to evolve faster in all cases (Fig. 5, left column).

**Figure 6:**
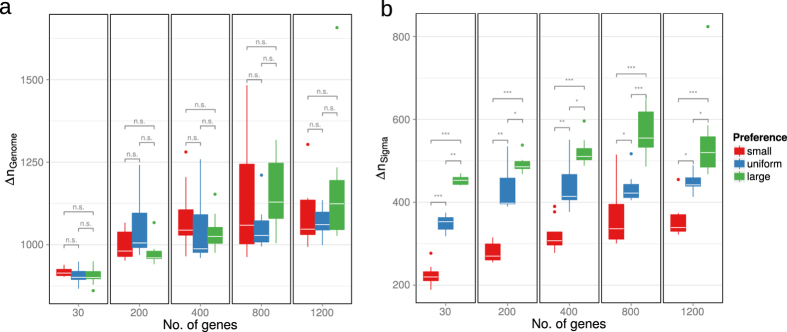
Generation number Δ*n* required to reach a threshold quality index of 0.01 in randomly generated transcription factors. (**a**) Evolutionary simulations were performed on the whole genome, (**b**) Evolutionary simulations were performed on the individual sigma factors. Bias in the choice of sigma factors is indicated by the color of the boxplots; red: small sigma factors were preferred in the generation of transcription factors, blue: genes were randomly chosen without bias, green: genes from large sigma factors were preferred. Asterisks indicate significance levels as determined by the Mann-Whitney test and corrected for multiple comparisons; ***p < 0.001, **p < 0.005, *p < 0.05, n.s.: not significant. Each boxplot represents results from simulations with ten different randomly generated transcription factors. Different regulon sizes were used as indicated on the horizontal axis.

Since the transcription factors studied here showed an enrichment of genes belonging to certain alternative sigma factors, the effect of a biased distribution of genes is of interest. Hence, the effects of a preference for genes from either large or small sigma factors were compared to a selection of all genes with equal probability. While there was no significant difference between regulons preferring small or large regulons and a random choice of genes with equal probabilities when the optimization was performed genomewide (Fig. 6a), a preference for small sigma factors led to significantly shorter evolution times in simulations optimizing within the sigma factors (Fig. 6b, red and blue boxplots). Similarly, the alternative sigma-factor evolution of regulons with genes chosen with a preference for large sigma factors was slower than in regulons with genes chosen according to a uniform probability distribution. While the effect is more pronounced in small regulons, the difference between the three cases was significant with all studied regulon sizes. Similar results are found when comparing the strength of the advantage that the sigma factor evolution provides over a genomewide optimization strategy (Fig. S4).

These results indicate that evolution in smaller, modular units is robustly advantageous compared to a genomewide evolution. This advantage, shown even for extreme corner cases, is likely to be relevant for the evolution of transcription factors independent of the size and sigma factor-usage of their regulon. Furthermore, comparing the different cases, it can be concluded that the evolution of transcription factors with small regulons and a bias towards small sigma factors might be more efficient. The latter finding pertains to the optimization of independent expression levels represented as a numerical vector, and the computer simulations do not take into account the functional relationship between genes grouped in common regulation units. In the evolution of real transcription factors, the optimal evolvability most likely represents a trade-off between structural, functional, and organizational concerns and the effect of a preference in sigma factors might be one of many factors that influence the speed and efficiency of evolutionary processes.

## Discussion

The analysis of the structural and operational state of the sigma factor network in the opportunistic pathogen *P. aeruginosa* uncovered a highly modular structure with only limited direct cross talk among alternative sigma factor regulons that are robustly activated in response to diverse forms of external stress^14^. This is important since the survival of living systems critically relies on the robustness of essential modules and their insensitivity to many environmental and genetic perturbations and thus cannot be radically altered without causing severe damage. The ability to evolve, however, requires genetic changes to adapt to new challenges. These changes might be achieved over many generations by altering the structure of the sigma factors und thus the function of the functional sigma factor modules. A second possibility to evolve is to alter the connections of functional modules in a way that enables the organism’s adaptation to new challenges^43^. There have been multiple reports on overlapping of regulons of the alternative sigma factor regulons with those of the house-keeping sigma factor RpoD in different bacterial species^45^,^46^,^47^. Here, we found that alternative sigma factor regulons are sensitive to the activity of transcription factors that functionally couple the regulons and significantly modulate transcription levels from the promoters of the alternative sigma factor regulons in the face of diverse environmental cues. Our finding that global transcription factors combine the functional modules of selected subsets of sigma factors indicate that evolution of connectivity is facilitated when resorting to the well proven, combat-ready alternative sigma factor functional modules that allow the cell to quickly adapt to new challenges and new stress conditions. With this our data support the view that widespread modularity of biological networks constitutes a key driver of evolvability^13^,^48^,^49^,^50^.

Remarkably, not only the speed of finding a newly evolved transcription factor in *in silico* testings could be increased further if their respective regulons were restricted to genes belonging to only a subset of the sigma factor regulons but also stability was significantly enhanced. The theoretical framework used for the *in silico* predictions relies on several simplifications. Most importantly, representation of gene expression targets as independent numerical values ignores functional relationships between genes, and the evolution of transcription factors as mutation and selection of a population of randomly chosen starting vectors simplifies and reduces a complex evolutionary process down to a well-known algorithm to make evolutionary simulations computationally feasible. Hence, only statements about structural advantages of a modular organization can be made and a detailed description of evolutionary parameters and comparisons with data on the evolution of transcription factors are beyond the scope of this article. However, the simulated evolution suggests a general advantage of modular systems and might apply to other examples of modular systems such as protein domains as well and is in line with the previously observed advantage of modularization in numerical optimization problems^51^,^52^,^53^. The robustness of the advantage in evolutionary efficiency in the predictions for the six measured transcription factors, and hypothetical transcription factors with different compositions underscores the fundamental role of a modular organization in evolutionary processes. These results clearly demonstrate that the modular structures of sigma factor regulons provide *P. aeruginosa* with a robust framework to function adequately in its environment and at the same time facilitate evolutionary change.

## Methods

### Strains

We selected PA14_19120 (*rhlR*, ID 37943), PA14_30650 (*gacA*, ID 34781), PA14_44490 (*anr*, ID 26855), PA14_50220 (*fleQ*, ID 41540), and PA14_62540 (*cbrB*, ID 44074) from the Harvard Medical School PA14 transposon mutant library^17^ as well as a deletion mutant of PA14_45960 (*lasR*) and recorded their transcriptional profiles. As a wild-type control we used the PA14_12430 (*ladS*, ID 38371) transposon mutant as the PA14 strain carries an inactivated *ladS* gene.

### mRNA profiling

RNA was isolated from two independent cultures (biological duplicates) of each of the *P. aeruginosa* PA14 transposon (*rhlR, gacA, anr, fleQ, cbrB*) and deletion (*lasR*) mutants. For each of those cultures three individual main cultures grown in LB medium at 37 °C with shaking at 180 rpm were pooled and cells were harvested at the early stationary phase (OD_600_ = 2.0). RNA extraction, cDNA library preparation and Illumina sequencing were performed as previously described^54^. Briefly, RNA protect buffer (Qiagen) was added to the harvested cells and RNA was isolated from cell pellets using the RNeasy plus kit (Qiagen). mRNA enrichment was performed using the MICROBExpress kit (Ambion). RNA was fragmented and ligated to RNA-adapters containing a hexameric barcode sequence for multiplexing. The resulting RNA-libraries were reverse transcribed and amplified resulting in cDNA libraries ready for sequencing. All but one sample were sequenced on an Illumina HiSeq 2500 device in the single-end mode, one sample was sequenced on an Illumina Genome Analyzer II-x. The raw and processed data are available at GEO under the accession number **GSE55328**. There we also provide a quality control report showing gene expression scatter plots, heatmaps of highly expressed genes and principal component analysis of the samples.

### Quantification of gene expression

The sequencing runs yielded between 3.1 × 10^6^ and 14.1 × 10^6^ single-end reads of 50 bp length, the sample sequenced on the Genome Analyzer II-x had read length of 36 bp. Sequence reads were separated according to their barcodes and barcode sequences were removed. Reads were mapped to the genome sequence of the reference strain *P. aeruginosa* PA14 wild-type using stampy^55^. More than 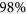 of the reads in each sample mapped to the PA14 genome. The R package DESeq^56^ was used for differential gene expression analysis. For each comparison, two biological replicates for each condition were used. The Benjamini and Hochberg correction was used to control the false-discovery rate (FDR) at 5% to determine the list of regulated genes. Genes were identified as differentially expressed if they were at least two-fold regulated in the mutant strain as compared to the *ladS* inactivated strain and their Benjamini-Hochberg corrected P value was maximally 0.05. The lists of differentially expressed genes are provided in the **GSE55328** archive.

Using the PseudoCAP annotation available for *P. aeruginosa* PA14, over- or under-representation was calculated by comparing normalized PseudoCAP classes experimentally detected and normalized PseudoCAP classes annotated using the following equation: EF = (number of specific PseudoCAP classes detected/number of all PseudoCAP classes detected)/(number of specific PseudoCAP classes annotated/number of all PseudoCAP classes annotated). As previously described^40^, an EF ≥ 1.5 was defined as overrepresentation and an EF ≤ 0.66 as underrepresentation.

### Hierarchical clustering

796 genes in PA14 have been defined to be highly variable under 14 environmental conditions^15^. The normalized read counts of those genes (*nRPKs*^15^) were used as an input for hierarchical clustering in R using the *hclust* function. We computed the pair-wise Pearson correlation between the normalized read counts for each gene under each condition and performed hierarchical gene clustering by progressively grouping them: at each step of the iterative algorithm the two genes or gene clusters that have the smallest distance were merged to form a new cluster, and two branches of a growing tree were joined. We used the average linkage rule; this means that the distance between two clusters is computed as the mean of all the distances between the genes in the first cluster and the genes in the second cluster. In the expression tree depicted in Fig. 1a the genes (vertical bars) are colored according to the alternative sigma factor they were exclusively assigned to^14^.

### *In silico* analysis of transcription factor evolution

A mathematical model of the evolution of transcription factors (TFs) on an unstructured genome is developed and compared to the same evolutionary process with a genome structured by alternative sigma factors. In this investigation, any functional relevance of alternative sigma factors is ignored. Further, the fact that TFs use only a small subset of alternative sigma factors is not considered unless stated otherwise. Any of these observations would increase the advantage of having alternative sigma factors such that this is a worst case investigation. However, the fact that newly evolved TFs do not simply take alternative sigma factors as they are but modulate the expression of each gene associated with the alternative sigma factors enters the investigation. The general idea of the simulation is to start from a vector of 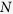 genes with each element representing an abstract expression level between 0 and 1 of this gene. A TF is represented as one such vector. Starting from 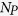 vectors a new target TF is evolved by an evolutionary algorithm. We use the following strategy:

1. An optimal TF for a new challenge is randomly defined by attribution of gene expression levels between 0 and 1 to each of the 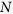 genes.
2. The target TF is (tried to be) evolved using two strategies:

- Genome-based: A set of NP randomly chosen TFs is defined by randomly choosing expression levels between 0 and 1 for all 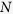 genes. These are evolved using the DE algorithm^43^.
- Alternative sigma factor-based: 11 alternative sigma factors with randomly attributed measured sizes are defined. These are mutually exclusive and cover the whole genome. For each of the alternative sigma factors, random gene expression levels between 0 and 1 are attributed to all genes associated with the respective alternative sigma factors. These are evolved using the DE algorithm^43^. The procedure is repeated for all alternative sigma factors.

1. The number of generations required to reach a success criterion is monitored in both evolution strategies. The success criterion is defined as the average per gene distance of the best TF in a generation from the target TF (denoted as *quality index*, QI) reaching values below 0.1%.

In addition, the CPU time used for both strategies is monitored, where in the alternative sigma factor-based strategy the time required for all alternative sigma factors is added up for comparison. Furthermore, the success of further evolution is controlled in both strategies by ensuring that the quality index decreases by at least 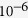 in 1000 generations. Otherwise, the evolution is stopped. The DE algorithm determines each parameter 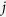 in the next generation TF 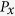 by attributing a mixture of three randomly chosen other TF vectors 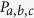 to this parameter value 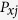. This is done with the probability 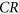 using the mutation function


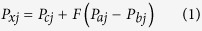


where 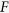 is a factor between 0 and 2 and 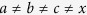. The parameters are varied and the presented simulations are performed with *CR* = 0.4 and *F* = 0.1. These parameters are most efficient in approaching the target TF with both, the alternative sigma factor-based and the genome-based strategy (Fig. S5). The number of evolved TF 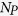 as well as the number of genes in the genome 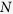 varied in order to determine how fast and how robustly the target TF is found. If applicable, the significance of the difference between both strategies in the success of TF evolution was evaluated by calculating 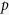 values from Fisher’s exact test in R. A robustness test in which a purely random mutation algorithm (*F* = 0) was used (Fig. S6) did not alter the conclusions.

### *In silico* predictions based on six real transcription factors and 150 hypothetical transcription factors with biased alternative sigma factor usage

To assess whether the theoretical predictions hold if real datasets instead of randomly generated transcription factors are used, the above simulation algorithm was used with the experimental data found for the six transcription factors *cbrB, gacA, anr, fleQ, rhlR, and lasR*. Instead of randomly generated values for the target vector, measured expression levels were used and normalized by the maximal expression level, resulting in target vectors with elements between 0 and 1. Since the evolution of transcription factors does not take place in isolation from the rest of the genome, all genes were included in the optimization algorithm and the algorithm was terminated accordingly. The quality index over all genes belonging to the regulon of the transcription factor of interest was monitored over all generations to keep track of its evolution. To further assess the influence of extremely biased usage of alternative sigma factors and different sizes of the transcription factors, this procedure was repeated with a number of hypothetical transcription factors. To generate these transcription factors, genes were first grouped according to the associated alternative sigma factors and then sorted by the size of the alternative sigma factors. Genes were then chosen at random either with uniform probability or with a probability given by a geometric distribution according to the size of associated alternative sigma factors, either in ascending or descending order, to introduce a strong bias towards small or large alternative sigma factors (Fig. S2). Transcription factors with regulons of 30, 200, 400, 800 and 1200 genes were generated by this procedure, leading to 15 different classes of transcription factors. For each of these classes, 10 transcription factors were generated and used as targets in evolutionary simulations. The number of generations, 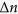, required to reach a threshold quality index of 0.01 was recorded for each simulation to compare the efficiency of the optimization process. The Wilcoxon-Mann-Whitney test was used for statistical comparison of 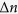 in different transcription factor classes and corrected for multiple comparisons according to the Holm-Bonferroni method.

## Additional Information

**How to cite this article**: Binder, S. C. *et al.* Functional modules of sigma factor regulons guarantee adaptability and evolvability. *Sci. Rep.* **6**, 22212; doi: 10.1038/srep22212 (2016).

## Supplementary Material

Supplementary Information
