# Supplementary material for: Functional modules of sigma factor regulons guarantee adaptability and evolvability
Source: Sci Rep. 2016 Feb 26;6:22212. doi: 10.1038/srep22212 (PMC4768184; doi:10.1038/srep22212)
Supplement: Supplementary Information [file srep22212-s1.pdf]

# SUPPLEMENTARY MATERIAL: Functional modules of sigma factor regulons guarantee adaptability and evolvability

**Sebastian C. Binder[1,+], Denitsa Eckweiler[2,3,+], Sebastian Schulz[2,3], Agata Bielecka[2,3], Tanja Nicolai[3], Raimo Franke[4], Susanne Häussler[2,3,+,*], and Michael Meyer-Hermann[1,5,+,*]**

[1]Department of Systems Immunology and Braunschweig Integrated Centre of Systems Biology, Helmholtz Centre for Infection Research, 38124 Braunschweig, Germany
[2]Institute for Molecular Bacteriology, TWINCORE GmbH, Center for Clinical and Experimental Infection Research, a joint venture of the Hannover Medical School and the Helmholtz Center for Infection Research, 30265 Hannover, Germany
[3]Department of Molecular Bacteriology, Helmholtz Centre for Infection Research, 38124 Braunschweig, Germany
[4]Department of Chemical Biology, Helmholtz Centre for Infection Research, 38124 Braunschweig, Germany
[5]Institute for Biochemistry, Biotechnology and Bioinformatics, Technische Universität Braunschweig, 38124 Braunschweig, Germany
[*]susanne.haeussler@helmholtz-hzi.de and mmh@theoretical-biology.de
[+]these authors contributed equally to this work

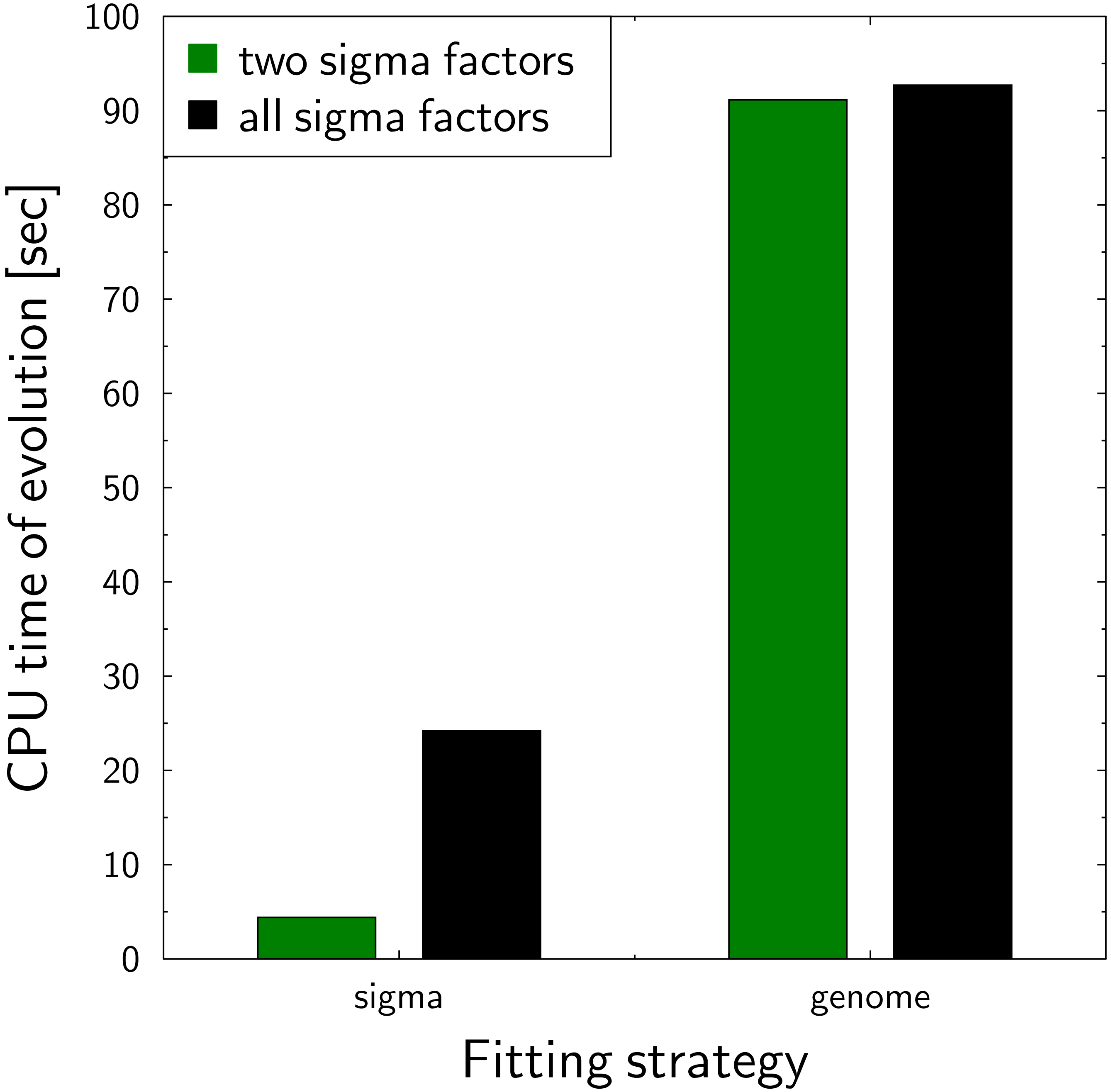


**Figure S1.** 1000 evolving vectors on a genome of 2000 genes with 11 sigma factors of random size were evolved based on the genes of the first two sigma factors only (green) which leads to substantially reduced search times in comparison to all sigma factors (black). The control times for evolution on the whole genome are provided for comparison. DE parameters: $CR = 0.4$ and $F = 0.1$.

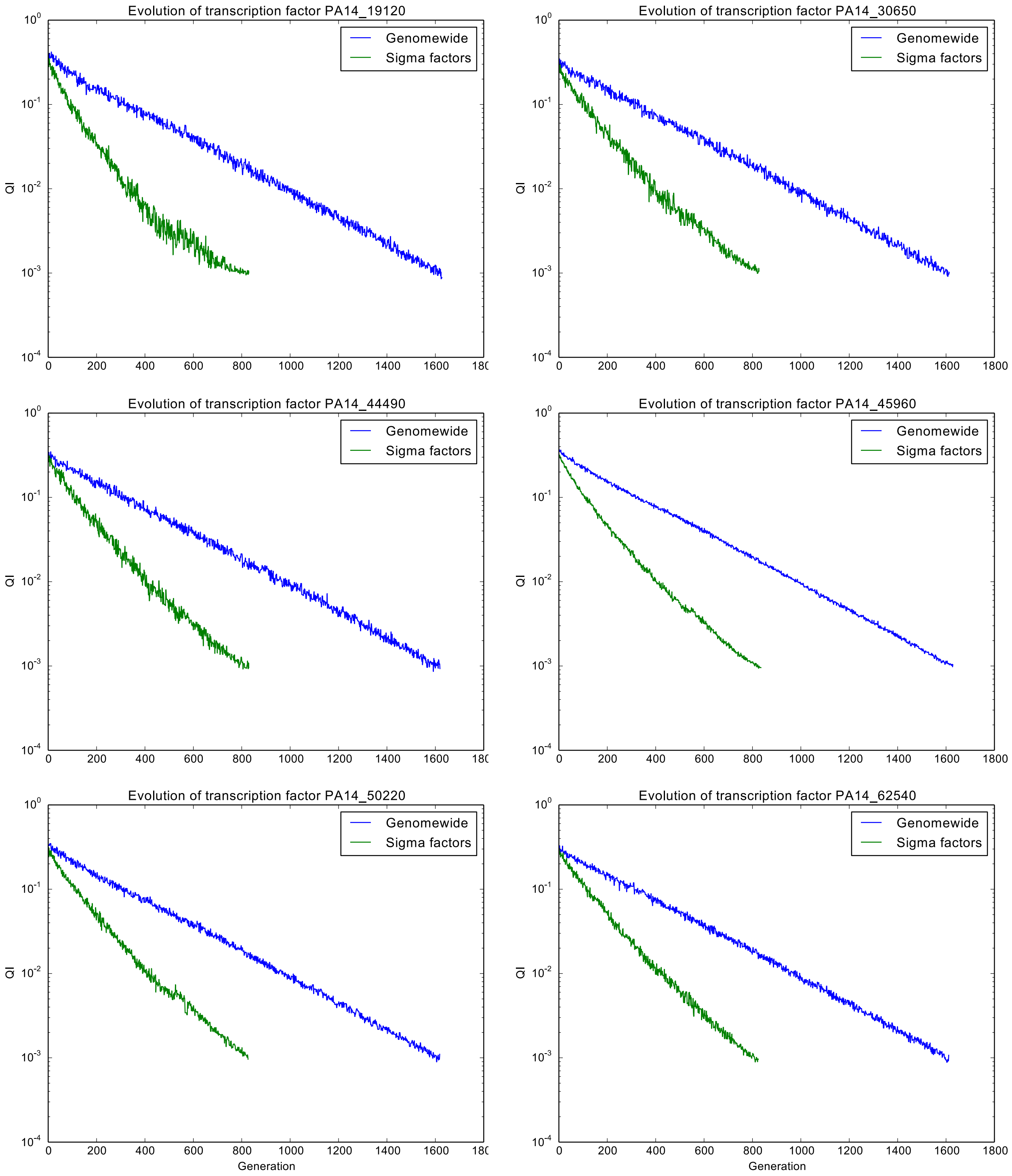


**Figure S2.** Evolutionary simulations with the experimentally determined transcription factor regulons. Evolution times to reach the termination criterion of a quality index of 0.001 were consistently shorter when optimization took place within single sigma factors instead of the whole genome.

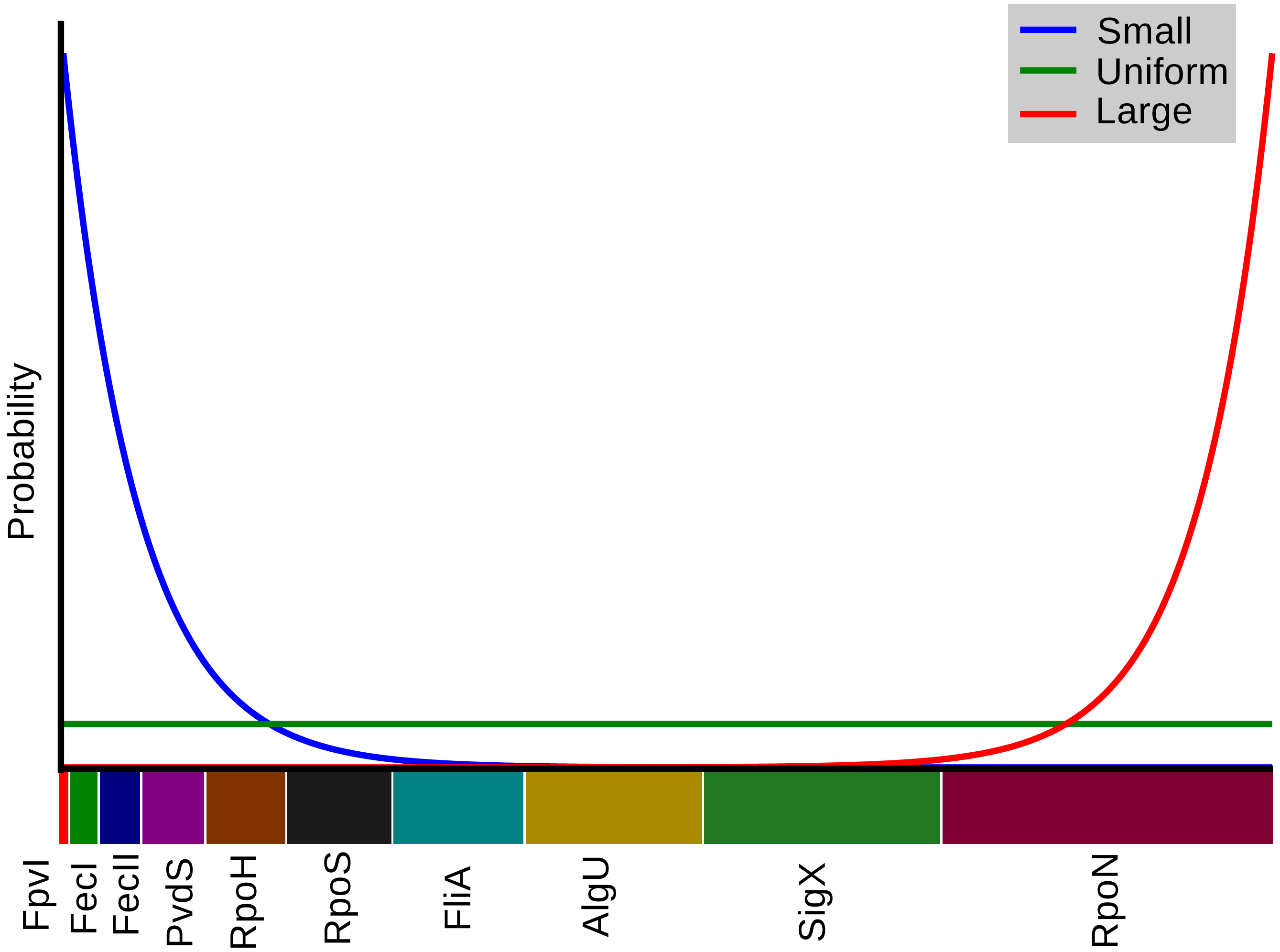


**Figure S3.** Generating synthetic transcription factors with different distributions. The illustration shows the selection of genes for the transcription factors. All genes were sorted according to the size of the corresponding sigma factors. In the random selection process to create the synthetic regulons, genes were selected according to the displayed probability functions: exponential distributions were used to prefer either genes from small (blue curve) or large (red curve) sigma factors. Both corner cases were compared to selecting all genes with equal probability (green line).

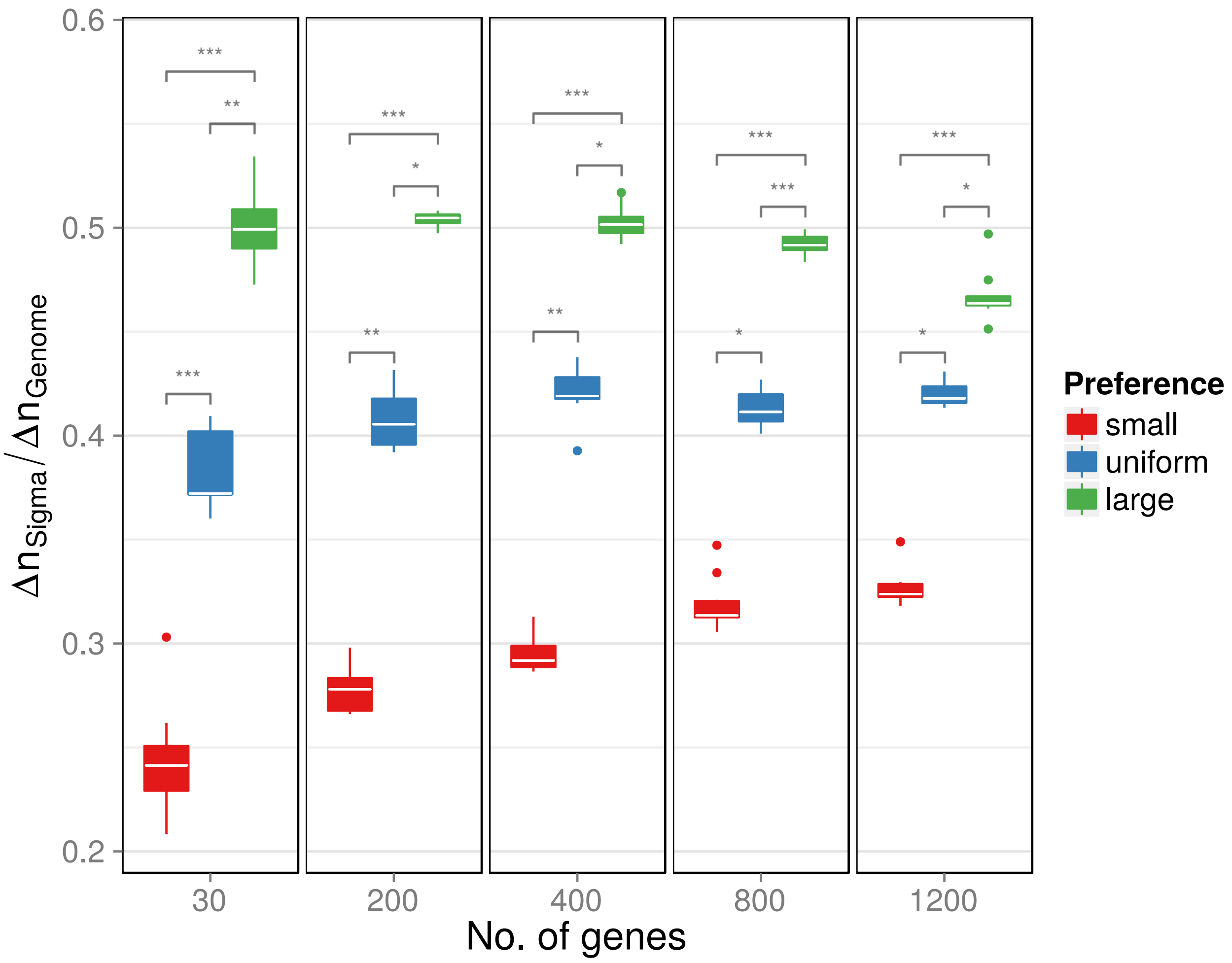


**Figure S4.** Advantage in evolutionary efficiency of the sigma factor evolution compared to a genomewide evolution strategy. The advantage is expressed as ratio of both strategies in the number of generations required to reach a threshold quality index of 0.01 in randomly generated transcription factors. Each boxplot represents results from simulations with ten different randomly generated transcription factors. Different regulon sizes were used as indicated on the horizontal axis. Bias in the choice of sigma factors is indicated by the color of the boxplots; red: small sigma factors were preferred in the generation of transcription factors, blue: genes were randomly chosen without bias, green: genes from large sigma factors were preferred. Asterisks indicate significance levels as determined by the Mann-Whitney test and corrected for multiple comparisons; ***: $p < 0.001$, **: $p < 0.005$, *: $p < 0.05$.

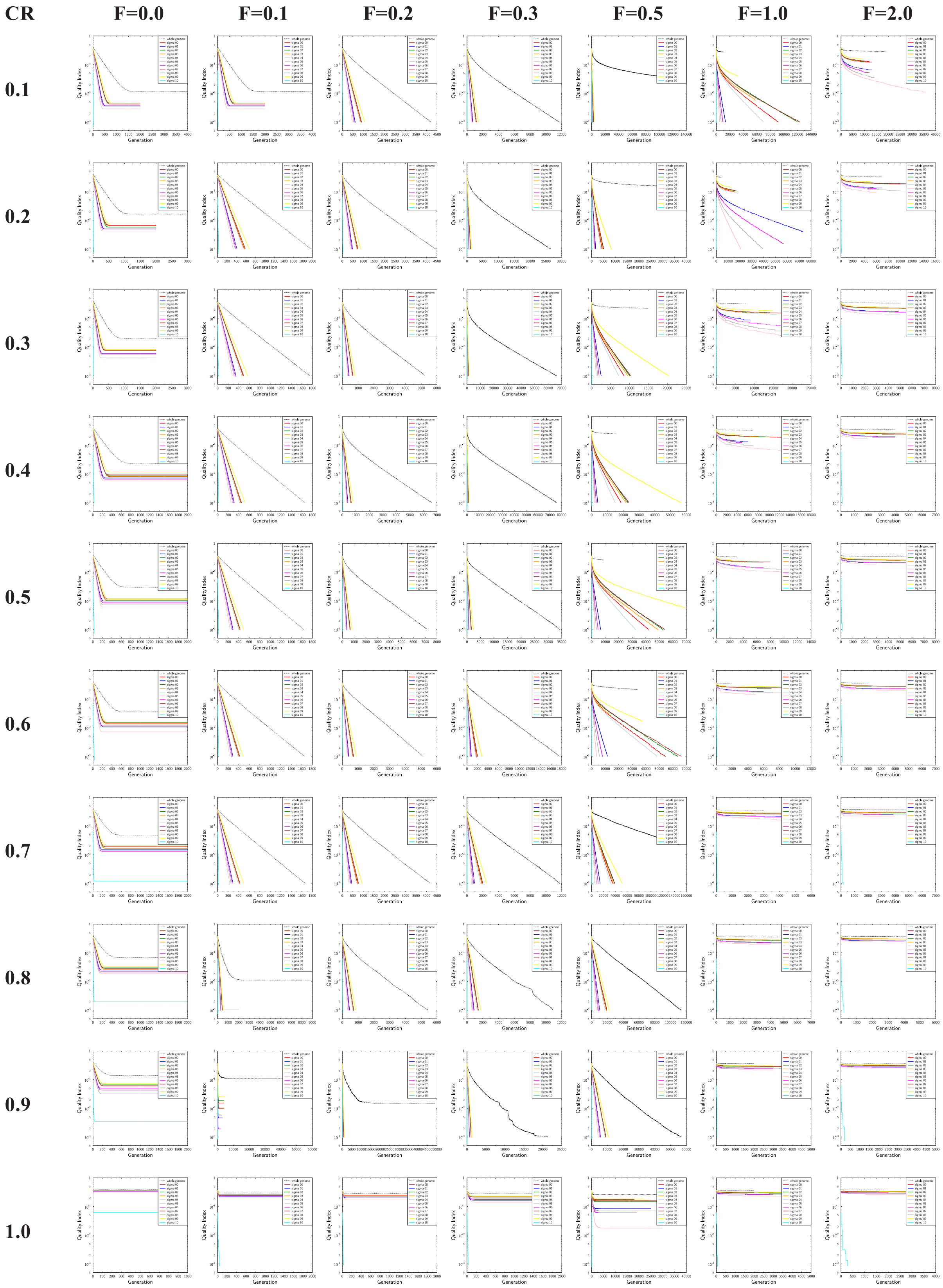


**Figure S5.** Scan of the Differential Evolution parameters. $F = 0.1$ and $CR$ between 0.3 and 0.7 was found to be the most effective range of optimizer parameters.

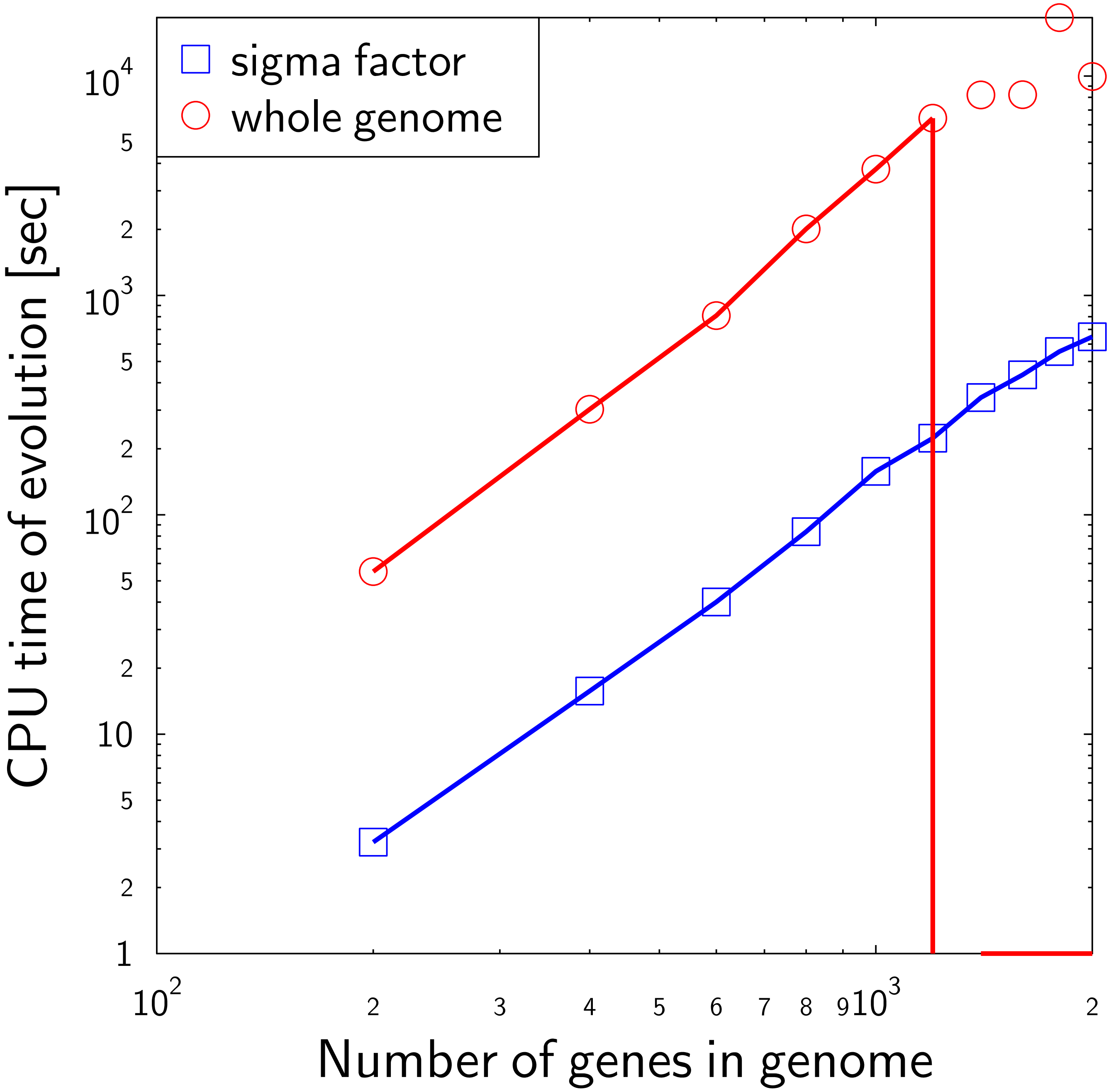


**Figure S6.** Evolution of a new TF *in silico* with a purely random algorithm yields qualitatively the same difference between genome-based and sigma-based evolution. However, the required number of generations to meet the success criterion was substantially larger. Note that for unsuccessful evolutions the CPU time was multiplied with zero which is visualized in the full lines. 2000 genes, 1000 evolved vectors with $CR = 0.001$ and $F = 0$. The threshold quality index for success was set 10-fold higher than in all other simulations in order to facilitate the search for the target TF.
